# The influence of different examiners on the Body Condition Score (BCS) in South American camelids—Experiences from a mixed llama and alpaca herd

**DOI:** 10.3389/fvets.2023.1126399

**Published:** 2023-02-03

**Authors:** Matthias Gerhard Wagener, Johannes Schregel, Nina Ossowski, Anna Trojakowska, Martin Ganter, Frederik Kiene

**Affiliations:** Clinic for Swine, Small Ruminants, Forensic Medicine and Ambulatory Service, University of Veterinary Medicine Hannover, Foundation, Hannover, Germany

**Keywords:** emaciation, clinical score, inter-rater reliability, nutrition, herd management, endoparasitosis, camelids

## Abstract

Particularly in unshorn llamas and alpacas with a dense fiber coat, changes in body condition often remain undetected for a long time. Manual palpation of the lumbar vertebrae is hence a simple and practical method for the objective assessment of body condition in South American camelids (SAC). Depending on tissue coverage, a body condition score (BCS) of 1 (emaciated) to 5 (obese) with an optimum of 3 is assigned. To date, there is a lack of detailed information on the comparability of the results when the BCS in llamas or alpacas is assessed by different examiners. Reliability of BCS assessment of 20 llamas and nine alpacas during a veterinary herd visit by six examiners was hence evaluated in this study. A gold standard BCS (gsBCS) was calculated from the results of the two most experienced examiners. The other examiners deviated by a maximum of 0.5 score points from the gsBCS in more than 80% of the animals. Inter-rater reliability statistics between the assessors were comparable to those in body condition scoring in sheep and cattle (*r* = 0.52–0.89; τ = 0.43–0.80; κ_w_ = 0.50–0.79). Agreements were higher among the more experienced assessors. Based on the results, the assessment of BCS in SAC by palpation of the lumbar vertebrae can be considered as a simple and reproducible method to reliably determine nutritional status in llamas and alpacas.

## 1. Introduction

The husbandry of South American camelids (SAC) is becoming more and more popular in Europe (1–4). In case of disease, llamas and alpacas are, however, often presented late for veterinary care. Hence, the animals are often severely emaciated or reveal anemia (5). In a recently published evaluation of 300 SAC presented to our clinic, we found that 60% of the alpacas and 70% of the llamas revealed a Body Condition Score (BCS) lower than the optimal score of three (5). At the same time, half of the SAC farms in Germany that participated in an online survey recently stated that they never had problems with emaciation. Furthermore, a quarter observed < 1 case of emaciation per year (1). This survey also showed that the occurrence of gastrointestinal endoparasitic infections and emaciation was more likely on farms with more animals than those with fewer animals (1). This discrepancy between the high amount of emaciated animals that are presented to the clinic and a rather low awareness of emaciation on the farms indicates that the assessment of the nutritional status is of particular importance in husbandry of SAC to recognize emaciation in time. Inadequate feeding management, chronic diseases, dental problems, and especially gastrointestinal endoparasites can lead to a poor nutritional status related to a low BCS (6–8). The decrease in body condition, sometimes within a relatively short space of time, is overlooked by the keeper due to the animal's dense fiber coat. In addition, SAC generally hide symptoms of disease for a long time and only display them at a very late stage (6). Visual examination alone is hence insufficient and may lead to incorrect results. When assessing the nutritional status of llamas and alpacas, manual palpation is vital (9). For the standardized assessment of the nutritional status in SAC, descriptions of a body condition score (BCS) from previous studies are available. Most of the authors recommend the palpatory examination of the lumbar spine for determining the BCS in SAC (9–16). However, depending on the source, other body regions, such as the thorax behind the elbow, the paralumbar fossa, or the area between the front and rear legs, are sometimes included in the assessment of the BCS of llamas and alpacas (6, 10, 12). In cattle, where the concept of body condition scoring is an important tool in herd management (17), several studies on the learnability and reproducibility of the BCS are available (18–21). Similar data can be found for sheep (22–24). To the best of our knowledge, accurate data on the comparability of BCS in llamas and alpacas are currently unavailable. In order to investigate the inter-rater reliability (25) for the BCS in SAC by palpation of the lumbar spine, we evaluated the results of six examiners with different levels of expertise assessing the BCS of llamas and alpacas during a herd visit in northern Germany.

## 2. Material and methods

### 2.1. Herd

The mixed llama and alpaca herd was located in northern Germany and had a size of 35 animals in early summer 2022. A total of five animals had died peracutely within a few weeks before the visit in August 2022. In addition, two crias had been born during the same period, resulting in a total of 32 animals (23 llamas and nine alpacas) at the time of our visit. The age of the animals ranged from 10 days to 19 years, all animals had been shorn between April and May 2022. The purpose of the visit was to check the health status of the remaining animals in the herd after the previously incurred losses. A clinical examination of each of the animals according to the routine protocol of the clinic was performed and the animals were vaccinated against clostridia. The BCS of the animals as part of the clinical examination was assessed by six examiners in order to increase the precision of the results and to obtain more routine in herd management of SAC. The assessment of the BCS is seen as a routine method in SAC husbandry, which should acclimatize the animals to stress-free handling (26). Since not all examiners assessed the BCS in three of the animals, these animals were excluded from the evaluation. Ultimately, body condition scores of 20 llamas and nine alpacas assessed by six examiners were included in this study.

### 2.2. Assessment of BCS

The BCS was assessed by palpation of the lumbar spine behind the last ribs according to previous descriptions (6, 9, 10) and ranged from 1 to 5 as follows:

BCS 1—emaciated
BCS 2—thin
BCS 3—optimal
BCS 4—overweight
BCS 5—obese

**Figure 1:**
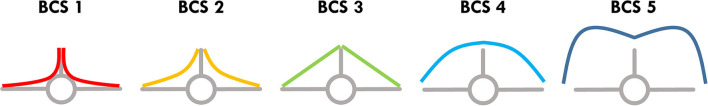
Schematic cross section through the lumbar spine. BCS in llamas and alpacas is assessed by palpating the tissue coverage of the lumbar vertebrae. The spinous and transverse processes as well as the connecting line between these two are palpated. If the BCS is optimal (3), this connecting line is straight; if this line is concave, the BCS is <3; if it is convex, the BCS is >3. Figure modified according to Wagener and Ganter (9).

All examiners palpated the spinous and transverse processes of the lumbar vertebrae as well as the muscle and fat coverage in between. In animals with an optimal nutritional status (BCS 3), the line between spinous and transverse processes should be neither convex nor concave (Figure 1). The more concave the line was, the lower the BCS was classified (BCS 1 and 2), the more convex the line was, the higher the BCS was classified (BCS 4 and 5). Steps of 0.5 in between were possible. Most of the animals were fixed in a chute for clinical examination. A few animals that were not compatible with the chute were restrained by only one person for the examination.

The results of the individual examiners for each animal were recorded as paper protocols on the farm and transferred to an Excel sheet (Microsoft Excel for Office 365) for further analysis later.

### 2.3. Examiners

The six different examiners had different levels of experience with body condition scoring in SAC:

- Examiner 1: veterinarian with more than 5 years of experience of regular practical assessment of BCS in SAC and small ruminants at clinic and herd level prior to the study
- Examiner 2: veterinarian with approx. one year of experience in regular practical assessment of BCS in SAC and small ruminants at clinic level prior to the study
- Examiner 3: veterinarian with approx. one year of experience in regular practical assessment of BCS in SAC and small ruminants at clinic level prior to the study
- Examiner 4: veterinarian with approx. one year of experience in regular practical assessment of BCS in small ruminants at herd level prior to the study
- Examiner 5: veterinary student who had learnt to assess BCS in SAC 3 years prior to the study
- Examiner 6: animal keeper, owner of the farm with more than 5 years of experience in regular practical assessment of BCS in SAC at herd level prior to the study.

### 2.4. Gold Standard BCS (gsBCS)

In order to obtain a “correct” BCS as a reference value for each animal, a gold standard BCS (gsBCS) was calculated for each animal according to Kleiböhmer et al. (19) who checked the accuracy of the BCS in cattle (19). Due to the experience and the close agreement of examiners 1 and 6, the gsBCS was calculated from their findings by calculating the means of both examiners for each animal. Examiners 1 and 6 both had more than 5 years of experience in determining the BCS. Examiner 6 tended to assess a lower BCS than examiner 1. In 16 animals, examiners 1 and 6 agreed, in seven animals, the BCS assessed by examiner 6 was 0.5 score points lower than that assessed by examiner 1 and in two animals, 1 score point lower than examiner 1. In four animals, examiner 6 was 0.5 score points higher than examiner 1. Since the two examiners differed by 0.5 score points for 11 animals, the calculated gsBCS for these animals resulted in 0.25 score points. Although these were mathematically correct, they did not represent a BCS that could be realistically examined. Therefore, for these animals, the BCS was rounded up or down to the nearest full score. For example, if the calculated value was 2.25, it was rounded down to 2, and if it was 2.75, it was rounded up to 3.

### 2.5. Statistical evaluation

Analysis of data was performed with Excel (Microsoft Excel for Office 365), SAS (SAS Enterprise Guide 7.1) and R [(R Foundation for Statistical Computing, Vienna, Austria, https://www.R-project.org) in combination with RStudio (Integrated Development for RStudio, Inc., http://www.rstudio.com)].

Descriptive statistics included mean, minimum and maximum of the BCS in each animal as well as mean, minimum and maximum of the assessed BCS by each examiner. In some of the groups examined, the values were not normally distributed. However, the mean was consistently used in the descriptive statistics, since some gradations were not visible in the median. In addition, the number of deviations from gsBCS were determined for each examiner by subtraction. For testing the inter-rater reliability of a BCS in ruminants, different statistical tests have been used in previously published studies (21, 22, 24, 27, 28). We used Spearman's rank correlation coefficient (r), Kendall's rank correlation coefficient (τ), and Cohen's weighted kappa (κ_w_) for testing pairwise correlation and agreement of the examiners with each other and with the gsBCS. In addition, one overall Kendall's coefficient of concordance (*W*) was computed, including only the examiners' scores without the gsBCS. Spearman's and Kendall's correlation was interpreted as follows: r/τ = 0–0.1: negligible correlation; r/τ = 0.1–0.39: weak correlation; r/τ = 0.4–0.69: moderate correlation; r/τ = 0.7–0.89: strong correlation; r/τ = 0.9–1.0: very strong correlation (29, 30). Cohen's weighted kappa and Kendall's coefficient of concordance were interpreted as follows: κ_w_/*W* = 0–0.2: slight agreement; κ_w_/*W* = 0.21–0.4: fair agreement; κ_w_/*W* = 0.41–0.6: moderate agreement; κ_w_/*W* = 0.61–0.8: substantial agreement; κ_w_/*W* = 0.81–1: almost perfect (31). Differences between llamas and alpacas were tested by using the unpaired two-samples Wilcoxon test. A *p*-value < 0.05 was considered significant.

## 3. Results

By the examination of 29 animals, in total, 174 BCSs were recorded. All assessed BCSs as well as further details on the animals can be found in Table 1. The mean value for all records was 3.29, the lowest BCS was 1.5, the highest 5. The gsBCS for all animals was 3.28 (mean) and ranged from 1.5 to 4. The alpacas of this herd had a lower gsBCS (mean: 2.83) than the llamas (mean: 3.48). However, the difference was not statistically significant (*p* = 0.08). The minimal BCS assessed by an examiner for all animals was 2.83 (mean) with a range of 1.5–4; the maximal BCS assessed by an examiner for all animals was 3.74 (mean) with a range of 2–5. The range of the BCSs that were assessed in an individual animal by the six examiners was 0.91 (mean) for all animals and was between 0 and 2 score points. In only one animal with a BCS of 4 did all six examiners give the same BCS. In 11 animals, the range of the examiners was 0.5 score points, the mean gsBCS in these animals was 3.00. In another 11 animals with a mean gsBCS of 3.18, the range was one score point. Four animals with a gsBCS of 3.75 (mean) had a range of 1.5 score points and two animals with a gsBCS of 4 each had a range of 2 score points in the BCS assessed by the six examiners.

**Table 1:** Overview of sex, age, assessed Body Condition Score (BCS) by each examiner, and calculated gsBCS (gold standard BCS) of the examined alpacas and llamas.

**Animal**	**Species**	Sex^*^	**Age (years)**	**Examiner 1**	**Examiner 2**	**Examiner 3**	**Examiner 4**	**Examiner 5**	**Examiner 6**	**gsBCS**
A 1	Alpaca	m	0	4	4	3.5	3.5	4	3.5	4
A 2	Alpaca	f	1	4	4	2.5	4.5	4	4	4
A 3	Alpaca	f	1	3.5	3.5	3	3.5	3.5	3.5	3.5
A 4	Alpaca	f	2	2.5	3	2.5	2.5	2.5	3	3
A 5	Alpaca	f	5	3.5	3.5	3.5	3	3	3.5	3.5
A 6	Alpaca	f	7	2.5	3	2.5	2.5	2.5	2	2
A 7	Alpaca	f	10	2	2	3	2.5	2.5	2	2
A 8	Alpaca	f	13	1.5	1.5	2	1.5	1.5	1.5	1.5
A 9	Alpaca	mn	13	2	2.5	2	2	2.5	2.5	2
L 1	Llama	m	1	3	3.5	3	3	2.5	3	3
L 2	Llama	f	2	3	4	4.5	3.5	3	3	3
L 3	Llama	f	2	3.5	3.5	3.5	4	4	3.5	3.5
L 4	Llama	f	3	3.5	3.5	4	4	3.5	4	4
L 5	Llama	m	4	3.5	3	4	3.5	3.5	3.5	3.5
L 6	Llama	f	5	3.5	4	3	4	3.5	3.5	3.5
L 7	Llama	mn	5	3.5	3.5	4	5	3	4	4
L 8	Llama	f	6	4.5	4.5	5	5	3.5	4	4
L 9	Llama	f	6	3	4	4	4	3.5	4	3.5
L 10	Llama	f	7	4	4	4	4.5	3.5	4	4
L 11	Llama	f	8	3	3	2.5	3	2.5	3	3
L 12	Llama	m	9	4	4	4	4	4	4	4
L 13	Llama	f	10	3.5	3	4	3.5	3.5	3.5	3.5
L 14	Llama	f	11	2.5	2	2.5	2.5	2.5	2.5	2.5
L 15	Llama	m	11	4.5	3.5	4	4	3.5	4	4
L 16	Llama	mn	14	3.5	3.5	4	3.5	2.5	4	4
L 17	Llama	f	14	3.5	3.5	4	5	4	4.5	4
L 18	Llama	mn	16	2.5	3	3	3	3	2.5	2.5
L 19	Llama	f	19	2	2	3	2	2	2.5	2
L 20	Llama	mn	19	3.5	3.5	3.5	3.5	3	4	4

^*^f, female; m, male; mn, male neutered.

Deviations of the individual examiners are displayed in Table 2. For five of the six examiners no significant difference could be detected between the examination of the BCS in alpacas and lamas regarding the deviations in the assessed BCS from the gsBCS (examiner 1: *p* = 0.38; examiner 2: *p* = 0.03; examiner 3: *p* = 0.21; examiner 4: *p* = 0.96; examiner 5: *p* = 0.74; examiner 6: *p* = 0.67).

**Table 2:** Overview of the results of each examiner and the number of assessed Body Condition Scores (BCS) that deviated from the gsBCS (gold standard BCS).

					**Deviation from gsBCS**			
	***n***	**Mean**	**Min-Max**	**Total score**	±**0.00** **BCS**	±**0.50** **BCS**	±**1.00** **BCS**	±**1.50** **BCS**
All examiners	174	3.29	1.5–5	573	92 (52.9%)	69 (39.7%)	10 (5.7%)	3 (1.7%)
gsBCS	29	3.28	1.5–4	95	0	0	0	0
Examiner 1	29	3.21	1.5–4.5	93	19 (65.5%)	10 (34.5%)	0 (0.0%)	0 (0.0%)
Examiner 2	29	3.29	1.5–4.5	95.5	12 (41.4%)	15 (51.7%)	2 (6.9%)	0 (0.0%)
Examiner 3	29	3.38	2–5	98	12 (41.4%)	12 (41.4%)	3 (10.3%)	2 (6.9%)
Examiner 4	29	3.45	1.5–5	100	12 (41.4%)	14 (48.3%)	3 (10.3%)	0 (0.0%)
Examiner 5	29	3.10	1.5–4	90	13 (44.8%)	13 (44.8%)	2 (6.9%)	1 (3.4%)
Examiner 6	29	3.33	1.5–4.5	96.5	24 (82.8%)	5 (17.2%)	0 (0.0%)	0 (0.0%)

Spearman's correlation analysis revealed strong significant correlations between gsBCS and examiners 2, 4, and 5 and moderate significant correlations between gsBCS and examiner 3. Interpreting the correlation and agreement between gsBCS and examiners 1 and 6 is unnecessary, since the gsBCS is the result of the assessments by examiners 1 and 6. Spearman's correlations between the individual examiners were almost all strong, moderate correlations were only found between examiner 3 and other examiners (1,2,5). The range for r between the individual examiners was 0.52–0.89.

When the same limits were applied to τ, Kendall's rank correlation coefficient resulted in weaker correlations. In this statistic, examiner 4 showed a strong correlation with the gsBCS and examiners 2, 3, and 5 a moderate correlation therewith. There was a moderate correlation among the individual examiners. A strong correlation was only found between examiner 1 and examiners 4, 5, and 6 as well as between examiner 4 and examiners 5 and 6. The range for τ between the individual examiners was 0.43–0.80.

Cohen's weighted kappa (κ_w_), on the other hand, showed better agreement than τ in most comparisons. The gsBCS had a substantial agreement with examiners 2–5. The kappa between examiners showed substantial agreement in almost all pairs except in the comparison of examiner 2 with examiners 3 and 5, and examiner 5 with examiners 3 and 4. The range for κ_w_ between the individual examiners was 0.50–0.79.

Kendall's coefficient of concordance (*W*) amounted to 0.78, which corresponded to an overall substantial agreement between the six examiners.

The exact values for Spearman's rank correlation coefficient, Kendall's rank correlation coefficient, and Cohen's weighted kappa are displayed in Tables 3, 4.

**Table 3:** Spearman's rank correlation coefficient (r), Kendall's rank correlation coefficient (τ), and Cohen's weighted kappa (κ_w_) are listed in bold.

**Spearman's rank correlation coefficient (r) Kendall's rank correlation coefficient (**τ**) Cohen's weighted kappa (**κ_**w**_***)***							
	**gsBCS**	**Examiner 1**	**Examiner 2**	**Examiner 3**	**Examiner 4**	**Examiner 5**	**Examiner 6**
gsBCS		* < 0.0001* * < 0.0001* –	* < 0.0001* * < 0.0001* –	* < 0.0001* *0.0002* –	* < 0.0001* * < 0.0001* –	* < 0.0001* * < 0.0001* –	* < 0.0001* * < 0.0001* –
Examiner 1	**0.91** **0.86** **0.86**		* < 0.0001* * < 0.0001* –	*0.0003* *0.0007* –	* < 0.0001* * < 0.0001* –	* < 0.0001* * < 0.0001* –	* < 0.0001* * < 0.0001* –
Examiner 2	**0.73** **0.65** **0.73**	**0.77** **0.69** **0.78**		*0.0007* *0.0009* –	* < 0.0001* * < 0.0001* –	* < 0.0001* * < 0.0001* –	* < 0.0001* * < 0.0001* –
Examiner 3	**0.66** **0.57** **0.66**	**0.62** **0.52** **0.64**	**0.59** **0.51** **0.58**		* < 0.0001* * < 0.0001* –	*0.0035* *0.0054* –	* < 0.0001* * < 0.0001* –
Examiner 4	**0.86** **0.78** **0.63**	**0.82** **0.73** **0.74**	**0.79** **0.69** **0.69**	**0.70** **0.61** **0.69**		* < 0.0001* * < 0.0001* –	* < 0.0001* * < 0.0001* –
Examiner 5	**0.73** **0.64** **0.71**	**0.80** **0.70** **0.74**	**0.70** **0.61** **0.50**	**0.52** **0.43** **0.54**	**0.81** **0.71** **0.54**		* < 0.0001* * < 0.0001* –
Examiner 6	**0.95** **0.92** **0.92**	**0.83** **0.74** **0.79**	**0.72** **0.62** **0.71**	**0.70** **0.60** **0.67**	**0.89** **0.80** **0.75**	**0.70** **0.60** **0.67**	

The first line in each cell represents Spearman's r, the second line Kendall's τ, and the third line Cohen's κ_w_. Respective *p*-values for Spearman's r and Kendall's τ are listed in italics. gsBCS, gold standard BCS.

**Table 4:** Agreements [Spearman's rank correlation coefficient (r), Kendall's rank correlation coefficient (τ) and Cohen's weighted kappa (κ_w_)] of all examiners with the gsBCS (line “gsBCS”; *n* = 6), and of the individual examiners with each of the other examiners (lines “examiner 1” to “examiner 6”; *n* = 5 each).

	***r*** =		τ =		κ_**w**_ ***=***	
	**Mean**±**SD**	**Min–Max**	**Mean**±**SD**	**Min–Max**	**Mean**±**SD**	**Min–max**
gsBCS	0.81 ± 0.12	0.66–0.95	0.74 ± 0.14	0.57–0.92	0.75 ± 0.11	0.63–0.92
Examiner 1	0.77 ± 0.09	0.62–0.83	0.68 ± 0.09	0.52–0.75	0.74 ± 0.06	0.64–0.79
Examiner 2	0.71 ± 0.08	0.59–0.79	0.62 ± 0.07	0.51–0.69	0.65 ± 0.11	0.50–0.78
Examiner 3	0.63 ± 0.08	0.52–0.70	0.53 ± 0.07	0.43–0.61	0.62 ± 0.06	0.54–0.69
Examiner 4	0.80 ± 0.07	0.70–0.89	0.71 ± 0.07	0.61–0.80	0.68 ± 0.08	0.54–0.74
Examiner 5	0.71 ± 0.12	0.52–0.81	0.61 ± 0.11	0.43–0.71	0.60 ± 0.10	0.50–0.74
Examiner 6	0.77 ± 0.09	0.70–0.89	0.67 ± 0.09	0.60–0.80	0.72 ± 0.05	0.67–0.79

## 4. Discussion and outlook

When considering the overall Kendall's coefficient of concordance, the agreement between the estimated BCSs of different examiners in this mixed herd of llamas and alpacas was surprisingly high. The largest range in BCS with 2 score points was found in only two of the animals, whereas the assessed BCS in 22 of the 29 animals (75.9%) differed only by a maximum of 1 score point between the examiners. When using the gsBCS as a definition of the correct score in each animal, only three (1.7%) of the 174 BCSs that were assessed in this study deviated from the gsBCS by more than 1 score point. Of course, this has to be considered within the context of the limitation that the gsBCS was calculated from the findings of examiners 1 and 6, and thus, there was already a close relationship between these three values. When interpreting the deviations from the gsBCS, the other examiners (2–5) did not deviate from the gsBCS in more than 40% of the animals, and did not deviate more than 0.5 score points in over 80% of the animals.

Examiners 1, 4, and 6 had the highest means for r, τ and κ_w_ compared to the other examiners. In contrast to the others, these examiners had more experience in assessing BCS in flocks. Furthermore, examiners 1 and 6 had the longest experience in assessing BCS in SAC. The other examiners who had clinical but not flock experience in assessing the BCS in SAC resulted in lower means for r, τ and κ_w_. In contrast to the SACs detected in the clinic, the SACs examined in this study had higher BCSs. The alpacas referred to our clinic revealed a BCS of 2.43 ± 0.77 (mean ± SD), the llamas a BCS of 2.20 ± 0.99 (mean ± SD) (5). This may have resulted in lower BCS being detected more reliably, and could be an explanation as to why animals with a higher gsBCS revealed a higher range of assessed BCS by the individual examiners. It is worth mentioning that the greatest differences in the estimation of the BCS between the examiners were in animals with a mean gsBCS of around 3, that represents an optimal nutritional status. The clinical consequences of these differences are therefore negligible.

Since no comparable studies for SAC are known so far, the results of studies in cattle and sheep were used for comparison. Kleiböhmer et al. (19) found that even inexperienced examiners who had received extensive training in BCS assessment were able to obtain reproducible BCS assessment results after 6 weeks (19). The 175 cows in their study were examined by 15 examiners. Herein, only 3% of the assessed BCS had a deviation of 0.5 score points from the gsBCS.

Other studies on the inter-rater reliability used a weighted kappa analysis for evaluation (21, 22, 24). In our study, the range of inter-rater reliability among examiners was κ_w_ = 0.50–0.79, which is comparable to other studies on the inter-rater reliability of the BCS in sheep or cattle.

Phythian et al. (22) investigated the inter-rater reliability for body condition scoring in sheep before and after a brief recalibration on the inter-observer agreement of three examiners (22). Before re-calibration, they found κ_w_ = 0.3–0.5 and *W* = 0.4–0.5, and thereafter, κ_w_ = 0.4–0.7 and *W* = 0.4–0.6. They also concluded that both a BCS as well in full as in half-unit scores can be determined by different examiners with a good agreement. In a study from New Zealand by Corner-Thomas et al. (24), BCSs of 45 sheep were assessed by both three experienced technicians and 23 farmers who had previously received training in BCS. Pairs of farmers revealed a higher variability in kappa (κ_w_ = 0.54–0.94) than the pairs of technicians (κ_w_ = 0.82–0.88) (24).

Kristensen et al. (21) tested the inter-rater reliability of 51 dairy veterinarians with different levels of experience after a workshop on BCS (21). The examiners assessed the BCS of 20 cows twice at an interval of 2.5 hours. The inter-rater reliability between the workshop participants was tested as well as the inter-rater reliability between participants and the six instructors who had also received a special training beforehand. That study showed that the inter-rater reliability of the second scoring showed better agreements (κ_w_ scoring 1 between workshop participants: κ_w_ = 0.50/0.17/0.78 [mean/minimum/maximum] κ_w_ scoring 2 between workshop participants: κ_w_ = 0.64/0.41/0.82). In addition, the respective pairs of workshop participants and instructors revealed a higher agreement than between workshop participants (κ_w_ scoring 1 between workshop participants and instructors: κ_w_ = 0.62/0.33/0.84 [mean/minimum/maximum]; κ_w_ scoring 2 between workshop participants: κ_w_ = 0.74/0.55/0.85) (21). This is also consistent with the findings from our study: examiners 1 and 6, who both had the longest experience in body condition scoring at SAC and could thus be compared with the instructors from the study by Kristensen et al. (21), had the highest kappa values compared to the other examiners.

However, when comparing the BCS in SAC to the BCS in cows, it is important to note that the BCS in cows involves multiple body regions, which enables a more precise awarding of 0.25 score points (18). In our study, where BCS was only assessed by palpation of the lumbar spine, such a precision cannot be achieved under practical conditions (9). The comparison to previous studies in sheep (22, 24), where the BCS was assessed in a similar manner, therefore seems more apt. The influence of different examiners concerning other body regions needs to be studied separately. This is supported by the findings of Zielke et al. (28) who found differences in the inter-rater reliability of BCS assessed in different body regions in bisons (28).

Since only inter-rater reliability of the BCS in SAC was evaluated in our study, intra-rater reliability has so far not been taken into account. The latter describes how reproducible the assessment of the BCS in an animal by the same examiner is. Intra-rater reliability of the BCS in cows and sheep has been studied by different research groups so far (20–24, 27, 32). Data on intra-rater reliability from ruminants suggest that more experienced examiners achieve higher kappa values than less experienced examiners (20, 21). This still remains to be tested for SAC. Kristensen et al. (21) also concluded that even limited training can lead to a significant improvement in validity and precision in the assessment of BCS (21).

Approaches for BCS assessment are not only available for the New World camelids but also for the Old World camelids in which different regions of the body, including the hump, are included (33–36). To date, there have been no studies on how reproducible the results are for assessing BCS in Old World camelids. Since the BCS could also provide an important indication of nutritional status and possible infections with gastrointestinal endoparasites in both New and Old World camels, the accuracy and repeatability of the BCS should also be investigated more closely in these species.

In conclusion, our findings indicate that the assessment of the BCS at the lumbar spine in SAC is a quite reproducible examination method, even when it is performed by different examiners. Our data as well as the results from other studies support the assumption that reproducibility increases with training and experience. If BCS is assessed regularly by staff involved in husbandry and veterinary care of SAC, emaciation as a sign of disease, stress, or lack of management can be detected at an early stage and appropriate measures of intervention can be taken in time.

## Data availability statement

The original contributions presented in the study are included in the article/supplementary material, further inquiries can be directed to the corresponding author.

## Ethics statement

Ethical review and approval were not required for the animal study because all data used for this study were collected during the clinical examination of the animals for diagnosis of a veterinary herd problem. Written informed consent was obtained from the owners for the participation of their animals in this study.

## Author contributions

MGW wrote the manuscript, the manuscript was reviewed by JS, NO, AT, FK, and MG, data were collected by MGW, JS, NO, AT, and FK, and data were evaluated by FK and MGW. The study was designed by MGW and supervised by MG and FK. All authors read and approved the final manuscript.
